# Feeling about living arrangements and associated health outcomes among older adults in India: a cross-sectional study

**DOI:** 10.1186/s12889-021-11342-2

**Published:** 2021-07-06

**Authors:** Shobhit Srivastava, Subhojit Shaw, Himanshu Chaurasia, Naina Purkayastha, T. Muhammad

**Affiliations:** 1 International Institute for Population Sciences, Mumbai, Maharashtra 400088 India; 2 National Institute for Research in Reproductive Health, ICMR, Parel, Mumbai, 400088 India; 3 Department of Statistics, Dibrugarh University, Dibrugarh, Assam India

**Keywords:** Dissatisfaction, Living arrangement, Health, Older adults, India

## Abstract

**Introduction:**

Advancement in the field of gerontology has been concerned with the well-being of older adults in a family setup that is associated with caregiving and support. While family life and well-being are defined by emotion, caregiving, and support activities, dissatisfaction/discontent with living arrangements is a public health concern, which is increasing with a rise in the proportion of the older population in the country. The study examines the association of dissatisfaction with living arrangements with health outcomes among older men and women in India.

**Methods:**

The present research used data from the 'Building a Knowledge Base on Population Aging in India'. The effective sample size for the analysis was 9181 older adults. Descriptive statistics and bivariate analysis were performed to present the preliminary estimates. For finding the association between various health outcomes over explanatory variables, binary logistic regression model was used separately for men and women.

**Results:**

About 22.8% of men and 30.8% of women who were living alone were dissatisfied with their present living arrangement. It was revealed that both men and women who were dissatisfied with their present living arrangements had significantly higher odds of experiencing poor self-rated health [OR:4.45, 3.25 ~ 6.09 and OR:3.32, 2.54 ~ 4.34], low psychological health [OR: 2.15, 1.61 ~ 2.86 and OR: 1.99, 1.57 ~ 2.53], low subjective well-being [OR: 3.37, 2.54 ~ 4.45 and OR: 3.03, 2.36 ~ 3.38], low ADL [OR: 1.77, 1.2 ~ 2.62 and OR: 1.59, 1.17 ~ 2.18, low IADL] [OR: 1.32, 1.03 ~ 1.69 and OR: 1.57, 1.24 ~ 1.98] and low cognitive ability [OR: 1.26, 0.98 ~ 1.61 and OR:1.44, 1.13 ~ 1.82] in comparison to their counterpart from men and women respectively.

**Conclusion:**

It is found that dissatisfaction with the living arrangement of older men and women is negatively associated with major health outcomes. Hence, appropriate policies and programs must be developed to promote increased family care and support and an improved residential environment that would create a feeling of comfort and happiness among older individuals.

**Supplementary Information:**

The online version contains supplementary material available at 10.1186/s12889-021-11342-2.

## Introduction

Since aging is usually associated with declining economic resources, decreasing cognitive ability, deteriorating physical health, and weakening social support [1], several studies have attempted to identify various correlates that influence different health outcomes among older adults. An important link between living arrangements and older adults’ health has been established suggesting that the living setup can affect levels and types of physical and psychological support older adults receive [2, 3]. However, the complexities of well-being at later phases may vary by the number of younger family members, differing emotional attachments, and caregiving [4, 5]. Further, researchers have found a positive association of family support with the self-rated health of older adults [6, 7]. A recent study also found that older adults who prefer a shared living arrangement among their adult children tend to report negative health outcomes [8]. Studies have also investigated the effects of the residential environment on the subjective well-being of older adults [9–12].

While the decline in co-residence with children reduced the familial support for older adults, as evidence suggests, it eventually led to increased psychological illnesses in later years of their lives [13–15]. Similarly, a substantive body of literature exists regarding the mediating role of familial support system between living arrangements and health of older people [16–19]. Kaufman, (2018) has found evidence of keeping a successful intimate partner that leads to improvement of all dimensional health [20]. In this regard, the enhanced familial ties have been perceived as an alternative for preventing negative health behaviors [21].

### Feeling about living arrangement and health status

Adequate care and support from the family, higher levels of satisfaction with living environments, and staying in their own home plays a crucial role in achieving successful aging [22, 23]. A voluminous body of literature exists regarding the factors related to satisfaction with single/co-residential living arrangements of older people [19, 24, 25]. Study shows that older persons who moved to a co-residential arrangement felt uncomfortable in the new environment due to their new status of having to depend on others for their day-to-day care needs [26]. Recent studies in India found that older people expressed satisfaction and a strong desire for remaining in their own homes and reported a sense of independence and autonomy in such living settings [3, 27].

Thus, advancement in the field of gerontology has been concerned with the feeling about the family setup that is associated with caregiving and support and the well-being of older adults. However, in India, studies that focused on feelings about living arrangements among older adults and their association with the health outcomes is scant. Moreover, since it is not the same to be ill (an objective condition) as it is to suffer from an illness (a subjective condition), objective health indicators cannot fully capture the richness of how families react to illness to reduce its impact on the wellbeing of the family [28, 29]. While assessing the health status of older adults, subjective wellbeing is also considered to be a key component [21]. Along with that, ADL and IADL are important tools to measure the overall health and wellbeing [30].

The purpose of the present study is to empirically examine the dissatisfaction with living arrangements and health outcomes among older men and women in India. Here, we hypothesize that the satisfaction with living arrangement is positively associated with better health outcomes among older adults.

## Methods

### Data

The present research used data from Building a Knowledge Base on Population Aging in India (BKPAI) which was National level survey and was conducted in 2011, across seven states of India. The survey was sponsored by Institute for social and economic change (ISEC), Tata Institute for social sciences (TISS), Institute for economic growth (IEG), and (United Nations Population Fund) UNFPA, New Delhi. The survey gathered information on various socio-economic and health aspects of aging among households of those aged 60 years and above. The data from seven states were collected which represents the various regions of India namely, North India, South India, Western India, and Eastern India [31].

**Fig. 1:**
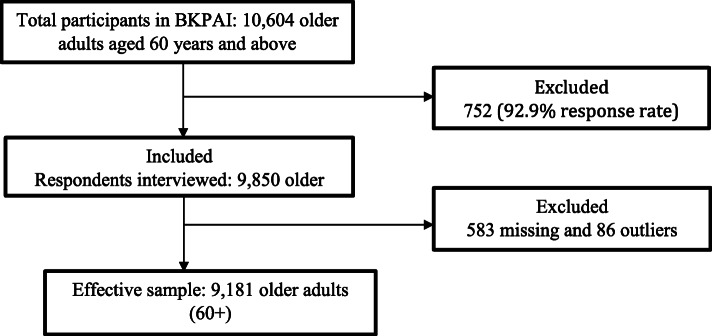
Sample selection for the present study

Being the survey of the older adults, the sample size was equally split between urban and rural areas, irrespective of the proportion of the urban and rural population. Eighty Primary Sampling Units (PSU) (villages or urban wards) – 40 urban and an equal number of rural – with 16 households per Primary Sampling Unit (PSU) having an older person were covered in the survey [31]. BKPAI collected information from 9850 older adults aged 60 years and above who were interviewed from 8329 households [31]. The sample included for the analysis after performing all pre-analysis procedures like dropping the missing data (583) and outliers (86 cases) was 9181 older adults (Fig. 1).

### Variable descriptions

#### Outcomes variables

The study analyzed six outcomes, collected through various tools that were previously used in the Indian context to categorize them in both continuous and binary forms. The six outcome variables were self-rated health, psychological health, subjective well-being (SWB), ability to do activities of daily living (ADL), ability to do instrumental activities of daily living (IADL), and cognitive ability.

The outcome variables were used in two forms. Firstly in the form of the index which was used to calculate mean by background characteristics and secondly in the form of the binary function to run binary logistic regression analysis. Self-rated health was having a scale of 1 to 5 “poor to excellent” and was categorized as 0 “good” (representing good, very good, and excellent) and 1 “poor” (representing poor or fair) [32]. Psychological health was having a scale of 0 to 12 based on experiencing stressful symptoms and was recoded as 0 “high” (representing 6+ scores) and 1 “low” (representing score 5 and less) [33, 34] (Cronbach Alpha: 0.90). Subjective wellbeing [8] was having a scale of 0 to 9 and was categorized as 0 “high” experiencing better experience (representing 6+ scores) and 1 “low” experiencing negative experience (representing score 5 and less) [35] (Cronbach Alpha: 0.89).

Ability to do activities of daily living was having a scale of 0 to 6 wherein it represents higher the score higher the independence. A score of was categorized as 0 “high” which represents full independence and 5 and less was categorized as 1 “low” which represents not fully independent to do activities of daily living (Cronbach Alpha: 0.93) [36]. The ability to do instrumental activities of daily living was having a scale of 0 to 8 representing higher the score higher the independence. A score of 6+ was categorized as 0 “high” representing high IADL and a score of 5 and less was recoded as 1 “low” representing low IADL [37, 38]. Cognitive ability was measured by the number of words recalled. To measure cognitive ability a scale of 0 to 10 was prepared representing higher the score better the cognitive ability. Five or more words were recoded as 0 “high” representing better cognitive ability and a score of four or less was recoded as 1 “low” representing low cognitive ability [39, 40]. The cut-offs for the outcome variables are based on the literature available and the (Additional file 1) contains all the questions and their recoding.

#### Explanatory variables

Feeling about the present living arrangement was assessed through the question “How do you feel about your present living arrangement” and was categorized as 0 “satisfactory” which includes comfortable and satisfactory and 1 “non-satisfactory” which includes not comfortable. Living arrangement was recoded as living alone, with the spouse, with children, and living with others. Age was categorized as 60–69, 70–79, and 80 + years. Sex was categorized as Men and Women. Educational status was categorized as no schooling, below 5 years of schooling, 6–10 years of schooling, and 11 and above years of schooling. Working status for the last year was categorized as no and yes. Marital status was categorized as not in union “included never married, widowed, divorced and separated” and currently in a union.

### Statistical analysis

Descriptive statistics and bivariate analysis were performed to present the preliminary estimates. For finding the association between various health outcomes over explanatory variables, the binary logistic regression model [41] was used separately for men and women. While applying logistic regression, outcome variables were recoded in binary forms (0 and 1). For instance self-rated health (0 “good” and 1 “poor”), psychological health (0 “high” and 1 “low”), subjective well-being (0 “high” and 1 “low”), IADL (0 “high” and 1 “low”), ADL (0 “high” and 1 “low”) and cognitive ability (0 “high” and 1 “low”). The main explanatory variable for the study was “feeling about present living arrangement” which was recoded as satisfactory and not-satisfactory.

The equation for logistic distribution is as follows:-

$$ \mathit{\ln}\left(\frac{\pi }{1-\pi}\right)=\alpha +{\beta}_1{X}_1+{\beta}_2{X}_2+{\beta}_3{X}_3\dots .{\beta}_n{X}_n $$

Where, *β*_0_, …. . , *β*_*M*_, are the regression coefficients indicating the relative effect of a particular explanatory variable on the outcome variables. These coefficients change as per the context in the analysis in the study. STATA 14 was used to carry out the analysis [42].

## Results

**Fig. 2:**
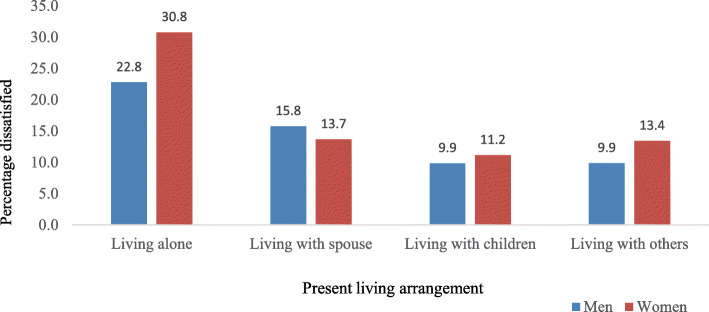
Percentage distribution of older men and women who were dissatisfied by their present living arrangement

Figure 2 depicts the percentage distribution of older adults who were dissatisfied with their present living arrangement, by sex. Findings from this figure suggested that, out of the total older adults living alone, the highest proportion of women reported dissatisfaction with their present living arrangement. Higher percentage of older men (22.8%) and women (30.8%) reported to be dissatisfied by their present living arrangement if they were living alone.

The characteristics of the target population in this study are summarized in Table 1. Around 63% of the older adults from the dataset belong to the age group of 60–69. More than half of the subjects under study were women. A total of 1050 (11.44%) older adults have reported their dissatisfaction with their living arrangements. Besides, around 6.06% of the older adults were living alone whereas around 14.58% of them were living with spouse only.

**Table 1:** Background characteristics of the study participants (*N* = 9181)

Background Variables	Sample	Percentage
**Poor SRH**		
No	4095	44.6
Yes	5086	55.4
**Low psychological health**		
No	7027	76.5
Yes	2154	23.5
**Low subjective well-being**		
No	6724	73.2
Yes	2457	26.8
**Low ADL**		
No	8503	92.6
Yes	678	7.4
**Low IADL**		
No	3998	43.6
Yes	5183	56.5
**Low cognitive ability**		
No	3670	40.0
Yes	5511	60.0
**Age (in years)**		
60–69	5815	63.3
70–79	2437	26.5
80+	929	10.1
**Sex**		
Men	4353	47.4
Women	4828	52.6
**Educational status (in years)**		
None	4186	45.6
Below 5 years	1886	20.5
6 to 10 years	2310	25.2
11+ years	799	8.7
**Working status**		
No	7076	77.1
Yes	2105	22.9
**Marital status**		
Not in union	3724	40.6
Currently in union	5457	59.4
**Feeling about present living arrangement**		
Satisfactory	8131	88.6
Non satisfactory	1050	11.4
**Living arrangement**		
Alone	546	6.0
with spouse	1462	21.9
With children	6480	70.6
Others	693	7.6
**Religion**		
Hindu	7389	80.5
Muslim	617	6.7
Sikh	771	8.4
Others	404	4.4
**Caste**		
Scheduled Caste	1802	19.6
Scheduled Tribe	470	5.1
Other Backward Class	3190	34.8
Others	3719	40.5
**Place of residence**		
Rural	4784	52.1
Urban	4397	47.9
**Wealth status**		
Poorest	1758	19.2
Poorer	1838	20.0
Middle	1823	19.9
Richer	1844	20.1
Richest	1918	20.9
**State**		
Kerala	1338	14.6
Himachal Pradesh	1452	15.8
Punjab	1249	13.6
West Bengal	1112	12.1
Orissa	1448	15.8
Maharashtra	1251	13.6
Tamil Nadu	1331	14.5
**Total**	9181	100

Table 2 depicts the mean scores for six different health conditions by background characteristic of the study population and the values closer to one indicated a poor state of health and vice versa. Findings from this study suggested that the older adults who were not satisfied with their current living arrangements acquired higher mean scores of different health conditions as compared to the ones who were quite satisfied with the same. The mean score for poor self-rated health, low psychological health, low subjective well-being, and low cognitive ability was higher among older adults living alone. However, the same for low ADL and low IADL was higher among older adults living with others. Findings also suggested that the mean scores for all the six different health conditions were higher among the oldest old of 80 years and above and lowest among older individuals belonging to the age group of 60–69 years. Apart from this, the mean scores for all six health conditions were higher among women, indicating that women were in a poor state of health and well-being than men.

**Table 2:** Mean scores for six different health conditions over background characteristics among older adults (*N* = 9181)

Variables	Poor self-rated health	Low psychological health	Low subjective well-being	Low ADL	Low IADL	Low cognitive ability
	Mean	Mean	Mean	Mean	Mean	Mean
**Feeling about present living arrangement**						
Satisfactory	0.52	0.20	0.22	0.07	0.55	0.58
Non satisfactory	0.81	0.47	0.63	0.12	0.70	0.74
**Living arrangement**						
Alone	0.57	0.33	0.40	0.03	0.36	0.63
With spouse	0.50	0.21	0.26	0.04	0.48	0.51
With children	0.56	0.23	0.25	0.08	0.60	0.61
Others	0.62	0.26	0.31	0.10	0.59	0.69
**Age (in years)**						
60–69	0.50	0.20	0.23	0.03	0.47	0.53
70–79	0.62	0.27	0.30	0.10	0.67	0.68
80+	0.70	0.35	0.39	0.25	0.86	0.79
**Sex**						
Men	0.52	0.21	0.24	0.06	0.56	0.53
Women	0.58	0.26	0.29	0.09	0.57	0.66
**Educational status (in years)**						
None	0.61	0.31	0.36	0.09	0.69	0.71
Below 5 years	0.57	0.22	0.24	0.08	0.51	0.64
6 to 10 years	0.45	0.13	0.14	0.04	0.40	0.41
11+ years	0.41	0.08	0.10	0.04	0.26	0.31
**Working status**						
No	0.58	0.25	0.28	0.09	0.61	0.62
Yes	0.47	0.20	0.24	0.01	0.43	0.53
**Marital status**						
Not in union	0.61	0.29	0.33	0.11	0.63	0.69
Currently in union	0.51	0.20	0.23	0.05	0.52	0.54
**Religion**						
Hindu	0.53	0.26	0.28	0.07	0.57	0.60
Muslim	0.67	0.23	0.30	0.12	0.57	0.67
Sikh	0.66	0.08	0.12	0.06	0.62	0.56
Others	0.61	0.16	0.22	0.07	0.31	0.64
**Caste**						
Scheduled Caste	0.60	0.28	0.34	0.08	0.63	0.66
Scheduled Tribe	0.46	0.32	0.35	0.06	0.64	0.71
Other Backward Class	0.53	0.26	0.28	0.08	0.53	0.56
Others	0.56	0.17	0.20	0.07	0.55	0.58
**Place of residence**						
Rural	0.57	0.25	0.28	0.08	0.59	0.63
Urban	0.51	0.19	0.22	0.07	0.48	0.52
**Wealth status**						
Poorest	0.62	0.37	0.47	0.08	0.67	0.71
Poorer	0.56	0.29	0.32	0.07	0.59	0.65
Middle	0.53	0.20	0.21	0.08	0.55	0.60
Richer	0.49	0.15	0.15	0.06	0.51	0.49
Richest	0.55	0.09	0.09	0.07	0.44	0.49
**State**						
Kerala	0.67	0.14	0.15	0.10	0.34	0.66
Himachal Pradesh	0.46	0.17	0.15	0.07	0.60	0.54
Punjab	0.67	0.07	0.11	0.05	0.60	0.55
West Bengal	0.78	0.29	0.48	0.11	0.69	0.82
Orissa	0.47	0.37	0.35	0.09	0.71	0.69
Maharashtra	0.41	0.23	0.34	0.04	0.48	0.55
Tamil Nadu	0.46	0.36	0.32	0.06	0.53	0.41
**India**	**0.55**	**0.23**	**0.27**	**0.07**	**0.56**	**0.60**

*Poor self-rated health (coded in binary form* i.e. *1 “poor or Fair” and 0 “Excellent, very good and good”)*

*Low psychological health: General Health Scale (coded in binary form* i.e. *1 “scores 5 or less” and 0 “scores more than 6”)*

*Low subjective well-being (coded in binary form* i.e. *1 “scores of 5 or less” and 0 “scores 6+”)*

*Low ADL: Activities of Daily living (coded in binary* i.e. *1 “scores of 5 or less” and 0 “scores 6+”)*

*Low IADL: Instrumental Activities of Daily living (coded in binary* i.e. *1 “scores of 5 or less” and 0 “scores 6+”)*

*Low cognitive ability (coded in binary* i.e. *1 “scores of 4 or less” and 0 “scores 5+”)*

The adjusted odds ratio (OR) for the relationship between living arrangements, their feeling about present living arrangements, and three different health conditions viz. poor self-rated health, low general health, and low subjective well-being, are shown in Table 3. Findings from this table suggested that both men and women who were dissatisfied with their present living arrangements acquired a higher chance of experiencing poor self-rated health, as compared to those who were satisfied with it, which has remained significant [OR:4.45, 3.25 ~ 6.09 and OR:3.32, 2.54 ~ 4.34] in the model. Also, older men and women who were dissatisfied with their living arrangement acquired almost two times higher odds [OR: 2.15, 1.61 ~ 2.86 and OR: 1.99, 1.57 ~ 2.53, *p*-value< 0.05] of having low psychological health than older adults who were satisfied with it.

**Table 3:** Logistic Regression estimates for poor self-rated health, low psychological health, and low subjective well-being among older adults (aged 60 years and above), sex-stratified

Variables	Poor self-rated health		Low psychological health		Low subjective well-being	
	OR (95% CI)	OR (95% CI)	OR (95% CI)	OR (95% CI)	OR (95% CI)	OR (95% CI)
	Men	Women	Men	Women	Men	Women
**Feeling about present living arrangement**						
Satisfactory	Ref.	Ref.	Ref.	Ref.	Ref.	Ref.
Non satisfactory	4.45*(3.25,6.09)	3.32*(2.54,4.34)	2.15*(1.61,2.86)	1.99*(1.57,2.53)	3.37*(2.54,4.45)	3.03*(2.36,3.88)
**Living arrangement**						
Alone	Ref.	Ref.	Ref.	Ref.	Ref.	Ref.
With spouse	1.17(0.65,2.09)	1.32(0.90,1.92)	0.47*(0.26,0.86)	0.78(0.50,1.21)	0.71(0.36,1.39)	1.15(0.76,1.74)
With children	1.40(0.80,2.44)	1.34*(1.01,1.79)	0.57(0.32,1.01)	1.13(0.83,1.52)	0.74(0.39,1.42)	1.08(0.80,1.46)
Others	1.31(0.70,2.46)	1.42(0.98,2.07)	0.91(0.47,1.75)	1.35(0.90,2.03)	1.12(0.54,2.33)	1.28(0.86,1.9)
**Age (in years)**						
60–69	Ref.	Ref.	Ref.	Ref.	Ref.	Ref.
70–79	1.23*(1.01,1.49)	1.12(0.93,1.36)	0.97(0.76,1.23)	1.24*(1.00,1.53)	0.92(0.73,1.17)	1.31*(1.06,1.60)
80+	1.22(0.88,1.67)	1.22(0.89,1.67)	1.29(0.92,1.83)	1.62*(1.19,2.2)	1.31(0.94,1.84)	1.78*(1.33,2.38)
**Educational status (in years)**						
No schooling	Ref.	Ref.	Ref.	Ref.	Ref.	Ref.
Below 5 years	0.99(0.79,1.24)	0.80(0.63,1.00)	0.73*(0.57,0.95)	0.74*(0.58,0.96)	0.70*(0.55,0.90)	0.63*(0.50,0.79)
6 to 10 years	0.08(0.63,1.01)	0.51*(0.39,0.65)	0.53*(0.39,0.71)	0.46*(0.32,0.67)	0.45*(0.34,0.60)	0.54*(0.39,0.73)
11+ years	0.55*(0.38,0.78)	0.45*(0.27,0.74)	0.42*(0.25,0.71)	0.44*(0.20,0.98)	0.43*(0.24,0.75)	0.59(0.31,1.15)
**Working status**						
No	Ref.	Ref.	Ref.	Ref.	Ref.	Ref.
Yes	0.86(0.71,1.04)	0.78(0.6,1.02)	0.77*(0.6,0.97)	0.87(0.65,1.17)	0.55*(0.43,0.69)	1.15(0.87,1.52)
**Marital status**						
Not in union	Ref.	Ref.	Ref.	Ref.	Ref.	Ref.
Currently in union	0.95(0.74,1.21)	0.94(0.77,1.14)	1.11(0.83,1.47)	1.01(0.8,1.27)	0.98(0.74,1.29)	0.91(0.73,1.14)
**Religion**						
Hindu	Ref.	Ref.	Ref.	Ref.	Ref.	Ref.
Muslim	1.17(0.81,1.68)	1.09(0.77,1.54)	1.17(0.76,1.79)	1.25(0.9,1.76)	1.40(0.89,2.19)	1.14(0.82,1.59)
Sikh	1.16(0.74,1.80)	0.87(0.58,1.29)	1.08(0.53,2.20)	0.90(0.46,1.76)	0.97(0.51,1.83)	1.37(0.81,2.33)
Others	1.07(0.7,1.66)	1.21(0.8,1.81)	0.85(0.48,1.50)	1.04(0.66,1.62)	1.2(0.65,2.2)	1.53*(1.03,2.26)
**Caste**						
Scheduled Caste	Ref.	Ref.	Ref.	Ref.	Ref.	Ref.
Scheduled Tribe	0.89(0.58,1.38)	0.82(0.55,1.21)	1.12(0.72,1.76)	0.8(0.54,1.17)	1.03(0.65,1.62)	0.93(0.63,1.37)
Other Backward Class	1.05(0.81,1.36)	1.14(0.89,1.48)	0.88(0.65,1.18)	0.71*(0.54,0.93)	0.96(0.71,1.3)	1.15(0.88,1.51)
Others	0.90(0.71,1.16)	1.15(0.91,1.47)	0.78(0.58,1.05)	0.83(0.64,1.09)	0.76(0.58,1.02)	0.89(0.69,1.15)
**Place of residence**						
Rural	Ref.	Ref.	Ref.	Ref.	Ref.	Ref.
Urban	0.98(0.81,1.17)	0.96(0.80,1.14)	0.95(0.75,1.20)	0.92(0.75,1.13)	1.20(0.96,1.51)	1.06(0.88,1.28)
**Wealth status**						
Poorest	Ref.	Ref.	Ref.	Ref.	Ref.	Ref.
Poorer	0.97(0.74,1.28)	0.74*(0.57,0.96)	1.15(0.86,1.53)	1.18(0.91,1.53)	0.97(0.74,1.27)	0.71*(0.55,0.92)
Middle	0.78(0.58,1.06)	0.57*(0.42,0.76)	0.95(0.68,1.33)	0.85(0.63,1.15)	0.64*(0.46,0.88)	0.55*(0.41,0.74)
Richer	0.71*(0.51,0.99)	0.47*(0.34,0.65)	0.77(0.51,1.15)	0.75(0.53,1.07)	0.38*(0.26,0.55)	0.49*(0.35,0.68)
Richest	0.70(0.48,1.03)	0.65*(0.45,0.93)	0.52*(0.31,0.87)	0.66*(0.43,1.00)	0.23*(0.13,0.38)	0.40*(0.27,0.58)
**State**						
Kerala	Ref.	Ref.	Ref.	Ref.	Ref.	Ref.
Himachal Pradesh	0.54*(0.38,0.78)	0.38*(0.27,0.55)	1.45(0.86,2.44)	1.03(0.67,1.57)	1.28(0.76,2.15)	0.94(0.61,1.45)
Punjab	1.15(0.75,1.76)	0.92(0.62,1.36)	0.64(0.31,1.32)	0.27*(0.15,0.48)	0.74(0.39,1.41)	0.64(0.39,1.08)
West Bengal	1.63*(1.13,2.37)	1.26(0.88,1.80)	2.60*(1.62,4.16)	1.29(0.90,1.85)	4.38*(2.83,6.79)	4.00*(2.81,5.68)
Orissa	0.35*(0.24,0.5)	0.2*(0.14,0.29)	3.72*(2.33,5.93)	2.12*(1.45,3.10)	2.06*(1.33,3.19)	1.49*(1.02,2.19)
Maharashtra	0.31*(0.23,0.43)	0.25*(0.18,0.34)	2.34*(1.52,3.62)	1.33(0.94,1.88)	2.34*(1.55,3.54)	2.62*(1.88,3.64)
Tamil Nadu	0.70*(0.49,0.98)	0.48*(0.35,0.66)	7.38*(4.57,11.92)	4.79*(3.32,6.91)	2.55*(1.63,3.99)	2.12*(1.48,3.04)

*Poor self-rated health (coded in binary form* i.e. *1 “poor or fair” and 0 “Excellent, very good and good”)*

*Low psychological health: General Health Scale (coded in binary form* i.e. *1 “scores 5 or less” and 0 “scores more than 6”)*

*Low subjective well-being (coded in binary form* i.e. *1 “scores of 5 or less” and 0 “scores 6+”)*

*if p < 0.05

*Ref* Reference, *OR* Odds Ratio, *CI* Confidence Interval

*The analysis is controlled for state level variations*

Similarly, older adults who were dissatisfied with their living arrangements were three times more likely to have low subjective well-being than their counterparts [OR: 3.37, 2.54 ~ 4.45 and OR: 3.03, 2.36 ~ 3.38]. Apart from this, women living with children were 34% more likely [OR: 1.34, 1.01 ~ 1.79, *p-*value< 0.05] to have poor self-rated health as compared to the older women living alone. However, in the case of men there lies no significant association with poor self-rated health. Men living with spouse only are less likely to have poor psychological health as compared to those living alone [OR: 0.47, 0.26 ~ 0.86, *p-*value< 0.05]. Older men aged 70–79 years acquired significantly higher odds [OR: 1.23, 1.01 ~ 1.49] of having poor self-rated health than the one belonging to the lower age group. Similarly, older women of age 70 years and above had a significantly higher chance of experiencing low psychological health and low subjective well-being, as compared to their counterparts. Both men and women with higher educational status had a lesser chance of experiencing poor self-rated health, low psychological health, and low subjective well-being as compared to the one with no formal education at all. Working men were less likely to have low psychological health and low subjective well-being [OR: 0.77, 0.6 ~ 0.97 and OR: 0.55, 0.43 ~ 0.69]. Apart from this, wealth status and states were also significantly associated with the occurrence of the three different health outcomes under study, respectively.

The adjusted ORs for the relationship between living arrangements, their feeling about present living arrangements, and three different health conditions viz. low ADL, low IADL, low cognitive ability are shown in Table 4. Findings suggested that older adults who were dissatisfied with their current living arrangement had higher odds of having low ADL [OR: 1.77, 1.2 ~ 2.62 and OR: 1.59, 1.17 ~ 2.18] and low IADL [OR: 1.32, 1.03 ~ 1.69 and OR: 1.57, 1.24 ~ 1.98] as compared to those who were satisfied with it, which has remained significant in the model. On considering the presence of low cognitive ability among the study subjects, findings showed that men were not significantly associated with the same, whereas women who were dissatisfied with their present living arrangement had higher odds [OR:1.44, 1.13 ~ 1.82] of having low cognitive ability in comparison to their counterparts. Older adults living with children or other family members were more likely to have low ADL [OR: 5.16, 1.2 ~ 22.18 and OR: 2.72, 1.63 ~ 4.51; OR: 5.18, 1.13 ~ 23.76 and OR: 2.87, 1.59 ~ 5.18] and low IADL [OR: 4.7, 2.91 ~ 7.59 and OR: 4.49, 3.43 ~ 5.87; OR: 4.11, 2.41 ~ 6.99) and OR: 3.9, 2.78 ~ 5.48] as compared to those who were living alone. Men and women living with a spouse had three times and two times [OR: 3.51, 2.13 ~ 5.76 and OR: 2.14, 1.51 ~ 3.02, *p-*value< 0.05] higher chance of having low IADL as compared to that of one living alone. Older adults including both men and women aged 80 years and above acquired significantly higher odds [OR: 4.21, 2.65 ~ 6.69 and OR: 4.50, 3.04 ~ 6.66, *p-*value< 0.05] of having poor self-rated health than the one belonging to the lower age group. Similarly, older women of age 70 years and above had a significantly higher chance of experiencing low ADL, low IADL, and low cognitive ability, as compared to the older adults belonging to the age group of 60–69, respectively. Both working men and women were less likely to have low ADL [OR: 0.29, 0.15 ~ 0.53 and OR: 0.14, 0.03 ~ 0.64, *p-*value< 0.05] and low IADL [OR: 0.46, 0.38 ~ 0.55 and OR: 0.49, 0.37 ~ 0.65,*p-*value< 0.05] as compared to their counterparts.

**Table 4:** Logistic Regression estimates for low ADL, low IADL and Low cognitive ability among older adults (aged 60 years and above), sex-stratified

Variables	Low ADL		Low IADL		Low cognitive ability	
	OR (95% CI)	OR (95% CI)	OR (95% CI)	OR (95% CI)	OR (95% CI)	OR (95% CI)
	Men	Women	Men	Women	Men	Women
**Feeling about present living arrangement**						
Satisfactory	Ref.	Ref.	Ref.	Ref.	Ref.	Ref.
Non satisfactory	1.77*(1.20,2.62)	1.59*(1.17,2.18)	1.32*(1.03,1.69)	1.57*(1.24,1.98)	1.26(0.98,1.61)	1.44*(1.13,1.82)
**Living arrangement**						
Alone	Ref.	Ref.	Ref.	Ref.	Ref.	Ref.
With spouse	3.74(0.84,16.59)	1.32(0.64,2.73)	3.51*(2.13,5.76)	2.14*(1.51,3.02)	0.75(0.47,1.21)	0.93(0.67,1.28)
With children	5.16*(1.2,22.18)	2.72*(1.63,4.51)	4.70*(2.91,7.59)	4.49*(3.43,5.87)	0.84(0.53,1.31)	1.20(0.93,1.54)
Others	5.18*(1.13,23.76)	2.87*(1.59,5.18)	4.11*(2.41,6.99)	3.90*(2.78,5.48)	0.89(0.54,1.48)	1.35(0.97,1.88)
**Age (in years)**						
60–69	Ref.	Ref.	Ref.	Ref.	Ref.	Ref.
70–79	1.83*(1.21,2.76)	1.93*(1.40,2.67)	1.37*(1.13,1.67)	2.16*(1.77,2.64)	1.33*(1.09,1.61)	1.51*(1.24,1.85)
80+	4.21*(2.65,6.69)	4.50*(3.04,6.66)	3.73*(2.63,5.30)	4.90*(3.44,6.96)	2.26*(1.64,3.12)	1.63*(1.18,2.24)
**Educational status (in years)**						
No schooling	Ref.	Ref.	Ref.	Ref.	Ref.	Ref.
Below 5 years	1.35(0.86,2.12)	0.72(0.48,1.1)	0.66*(0.53,0.83)	0.53*(0.43,0.66)	0.84(0.67,1.06)	0.65*(0.52,0.81)
6 to 10 Years	1.39(0.83,2.35)	0.96(0.55,1.66)	0.48*(0.38,0.61)	0.26*(0.2,0.35)	0.4*(0.32,0.51)	0.40*(0.31,0.51)
11+ years	1.30(0.50,3.37)	1.00(0.41,2.42)	0.20*(0.14,0.30)	0.19*(0.11,0.31)	0.25*(0.18,0.36)	0.20*(0.12,0.31)
**Working status**						
No	Ref.	Ref.	Ref.	Ref.	Ref.	Ref.
Yes	0.29*(0.15,0.53)	0.14*(0.03,0.64)	0.46*(0.38,0.55)	0.49*(0.37,0.65)	0.77*(0.64,0.92)	1.05(0.81,1.37)
**Marital status**						
Not in union	Ref.	Ref.	Ref.	Ref.	Ref.	Ref.
Currently in union	1.09(0.70,1.69)	1.26(0.88,1.81)	0.89(0.70,1.14)	0.82(0.67,1.01)	0.87(0.68,1.11)	0.97(0.81,1.18)
**Religion**						
Hindu	Ref.	Ref.	Ref.	Ref.	Ref.	Ref.
Muslim	1.61(0.85,3.02)	1.65*(1.05,2.57)	1.22(0.86,1.75)	1.65*(1.19,2.28)	0.99(0.70,1.40)	0.87(0.61,1.23)
Sikh	1.64(0.67,4.05)	0.97(0.48,1.95)	1.12(0.74,1.69)	0.94(0.63,1.42)	1.02(0.68,1.54)	1.03(0.70,1.50)
Others	0.84(0.30,2.35)	0.80(0.45,1.43)	0.5*(0.32,0.79)	1.03(0.67,1.57)	1.24(0.83,1.85)	1.53*(1.04,2.24)
**Caste**						
Scheduled Caste	Ref.	Ref.	Ref.	Ref.	Ref.	Ref.
Scheduled Tribe	0.87(0.38,2.00)	0.85(0.38,1.86)	1.21(0.79,1.86)	0.74(0.49,1.11)	1.49(0.98,2.27)	1.09(0.72,1.66)
Other Backward Class	1.22(0.75,1.98)	0.88(0.58,1.35)	1.16(0.90,1.49)	0.89(0.68,1.16)	0.90(0.70,1.16)	1.23(0.95,1.59)
Others	0.66(0.40,1.09)	1.15(0.78,1.70)	1.13(0.89,1.44)	0.90(0.71,1.16)	0.85(0.67,1.08)	1.01(0.79,1.30)
**Place of residence**						
Rural	Ref.	Ref.	Ref.	Ref.	Ref.	Ref.
Urban	0.85(0.58,1.26)	1.17(0.87,1.57)	1.17(0.97,1.40)	0.83*(0.69,0.99)	1.06(0.88,1.27)	0.96(0.81,1.13)
**Wealth status**						
Poorest	Ref.	Ref.	Ref.	Ref.	Ref.	Ref.
Poorer	0.85(0.49,1.5)	1.14(0.73,1.76)	0.73*(0.56,0.96)	1.04(0.79,1.37)	0.93(0.71,1.21)	1.1(0.84,1.44)
Middle	1.13(0.6,2.11)	0.88(0.54,1.45)	0.77(0.57,1.04)	0.80(0.59,1.09)	0.77(0.57,1.04)	0.94(0.70,1.26)
Richer	0.66(0.31,1.41)	0.81(0.47,1.39)	0.60*(0.44,0.83)	0.72*(0.52,1.00)	0.51*(0.37,0.71)	0.65*(0.47,0.89)
Richest	0.84(0.37,1.91)	0.76(0.42,1.39)	0.48*(0.33,0.69)	1.10(0.76,1.60)	0.56*(0.39,0.81)	0.70*(0.49,0.99)
**State**						
Kerala	Ref.	Ref.	Ref.	Ref.	Ref.	Ref.
Himachal Pradesh	1.67(0.80,3.47)	0.73(0.41,1.31)	2.38*(1.64,3.44)	3.52*(2.46,5.04)	0.38*(0.27,0.53)	0.59*(0.41,0.85)
Punjab	0.77(0.30,1.96)	0.38*(0.20,0.72)	2.51*(1.64,3.85)	3.03*(2.00,4.57)	0.46*(0.30,0.70)	0.46*(0.31,0.67)
West Bengal	2.92*(1.43,5.95)	0.85(0.51,1.41)	3.08*(2.15,4.41)	6.48*(4.60,9.12)	1.72*(1.19,2.48)	2.44*(1.63,3.64)
Orissa	1.81(0.88,3.72)	0.64(0.35,1.19)	2.71*(1.89,3.89)	6.18*(4.29,8.90)	0.6*(0.42,0.85)	0.62*(0.43,0.89)
Maharashtra	1.53(0.76,3.09)	0.26*(0.14,0.48)	1.29(0.93,1.79)	2.84*(2.08,3.89)	0.38*(0.28,0.52)	0.49*(0.37,0.66)
Tamil Nadu	1.96(0.85,4.51)	1.24(0.73,2.11)	3.79*(2.64,5.43)	2.38*(1.72,3.29)	0.27*(0.19,0.39)	0.30*(0.22,0.42)

*Low ADL: Activities of Daily living (coded in binary* i.e. *1 “scores of 5 or less” and 0 “scores 6+”)*

*Low IADL: Instrumental Activities of Daily living (coded in binary* i.e. *1 “scores of 5 or less” and 0 “scores 6+”)*

*Low cognitive ability (coded in binary* i.e. *1 “scores of 4 or less” and 0 “scores 5+”)*

*if p < 0.05

*Ref* Reference, *OR* Odds Ratio, *CI* Confidence Interval

*The analysis is controlled for state level variations*

## Discussion

The present study explored how the dissatisfaction with living arrangements of older men and women affects their health. It is found that a large number of older adults (12.5%) are dissatisfied with their current living arrangement especially, those older adults who live entirely on their own and it is significantly associated with negative health outcomes. The findings are consistent with previous studies suggesting that living alone is a significant predictor of reporting problems on mobility, pain/discomfort, and anxiety/depression [43]. Results demonstrate that the co-residential living arrangement is positively associated with older people’s satisfaction. The results also show that the satisfaction of the older men and women is low when they live alone, but they are satisfied when they live with their spouses or children, or others. In such cases, independent children and society need to encourage the older parents to get married again for late-life support and well-being.

A wealth of studies have shown that living arrangements are critical to health in old age [44–47]. Results from this study also show that older people’s dissatisfaction with living arrangements is closely related to adverse health outcomes with several indicators of health, SWB, ADL, IADL, and cognitive ability among the older adults in India. We found that in comparison with men and those living alone, women living with a spouse /others were more likely to report having excellent or very good health. Though studies have shown that having family members’ support reduces the effect of any type of problems on older adults and it gives them the strength and positive mental attitude to handle their daily lives [7, 21, 48]. On the other hand, previous studies on older adults living alone showed that they often have better health than those living with spouse and others which is true for older adults who can live alone by choice with sufficient resources [49–51]. However, when asked about the feeling of their living arrangement our study found that they were not satisfactory. This finding indicates that living alone, among older adults, may be a marker for improved health and greater functional independence.

Previous studies found that mental health status was good for those living with family members (spouse or children) compared with older adults living alone [52–54]. Consistently, our findings showed that men living alone had low psychological health while the association was insignificant in the case of women, though when functional health was considered in relation with their living arrangements, findings showed that both men and women co-residing with spouse or children or others had lower ADL and IADL functioning in comparison to those lived alone with higher odds among older men. Thus, the gender variations we observed between living arrangements and health status are also worth debating. One study conducted in the United States found that living alone can be beneficial for the SWB of older people [55].

Furthermore, the current finding that older men and women in the present study who lived alone were more likely to have better outcomes in ADL and IADL compared to those who lived with spouse and children was in variance with multiple studies that showed various health disadvantages of living alone, for example, a recent study reported that the occurrence of functional disabilities among older individuals was largely associated with lack of relatives and social contacts [56]. Nevertheless, consistent with our finding, studies showed similar results [57, 58], suggesting that those who lived alone were less likely to encounter ADL decline at baseline, thus indicating a need for further investigation on this particular area. Moreover, there was no significant difference found in the present study between men and women in their cognitive health associated with the living arrangement. Concordantly, while many reported no significant differences [59, 60], few studies stated significant gender-based differences in cognition and psychological wellbeing due to factors such as widowhood and lack financial support [61–64].

In summary, consistent with some recent micro-level work on population aging in India that has found that living arrangements and care and support for older adults are a core determinant of their health status [47, 65–67], the results of this study also showed that living arrangements and feeling about living arrangement played a significant role in predicting health outcomes in older adult life.

Our findings, as with any study, should be considered in light of their potential limitations. We depend on secondary cross-sectional data analysis which is unable to address reverse causality issues. However, findings based on self-reported data are subject to recall bias and reporting errors are possible, and therefore a single dependence on these responses could not adequately capture the relationship between the older adults’ living condition and its related health conditions. However, the study has a large sample size with a good response rate, that advances knowledge on living arrangements and health status of the older adults in a culture that values the interdependence of the family; a cultural context that is widely predominant in developing countries.

## Conclusion

The present study shows that, in terms of self-reported health status, general health, and subjective well-being, older people living alone and dissatisfied with their current living have poor health. Since filial obligations can play a pivotal role in reducing the discontent among older people, it is important to strengthen the values in care and support and enhance the capacity of the families providing incentives to minimize the financial and physical hardships of family members.

Further, the policymakers and health care professionals can engage those who are living alone with poor health status by allocating the resources that could target caregivers and close residents. Such efforts might include civic and community programming, and accessible and integrated models of health care delivery that address the problems of older individuals. The current evidence also suggests that further investigation is needed on why specific arrangements might be beneficial for health of the older individuals, so that other configurations of support can be developed such as institutional ones where both SWB and ADLs can be improved for both men and women and eliminate the intergenerational conflicts and gender differentials in caregiving.

## Supplementary Information

**Additional file 1. **Appendix file.

## Data Availability

The dataset generated and/or analysed during the current study is available in the repository, and accessible on request through http://www.isec.ac.in/

## Abbreviations

ADL: Activities of daily living
IADL: Instrumental activities of daily living
SRH: Self-rated health
BKPAI: Building a Knowledge Base on Population Aging in India
OR: Odds Ratio
CI: Confidence Interval
