# Supplementary material for: Feeling about living arrangements and associated health outcomes among older adults in India: a cross-sectional study
Source: BMC Public Health. 2021 Jul 6;21:1322. doi: 10.1186/s12889-021-11342-2 (PMC8258997; doi:10.1186/s12889-021-11342-2)
Supplement: Supplementary file 1 — Additional file 1. Appendix file. [file 12889_2021_11342_MOESM1_ESM.docx]

**Appendix file**

**Type of ADL**

1. Bathing
2. Dressing
3. Toilet
4. Mobility
5. Continence
6. Feeding

The response were: 1. Do not require assistance……0

2. Required partial assistance….1

3. Required full assistance……..1

Now I am going to ask you some questions regarding instrumental activities of daily living (IADLs) which are activities that are not necessary for basic functioning of daily life, but they let an individual live independently in a community.

**Types of IADL**

1. Ability to use telephone
2. Shopping
3. Food preparation
4. Housekeeping
5. Laundry
6. Transportation
7. Medication
8. Finances

The response were:

Operates phone on own initiative----1

Dials a few well known numbers-----1

Answers the phone but does not dial--0

Cannot use phone------------------------0

Takes care of all shopping needs independently--------1

Shops independently for small purchases----------------0

Needs to be accompanied on any shopping trip---------0

Completely unable to shop---------------------------------0

Plans, prepares and serves adequate meals independently---1

Prepares adequately means if supplied with ingredients------0

Heats, serves meals; does not maintain adequate diet---------0

Needs to have meals prepared and served----------------------0

Maintains house alone or with help for heavy work----------1

Performs light daily tasks e.g. dish washing, bed making----1

Performs light daily tasks but cannot maintain cleanliness----1

Needs help with all home maintenance tasks---------------------1

Does not participate in any housekeeping tasks------------------0

Does personal laundry completely ................ 1

Launders small items, rinses socks, etc. ............... 1

All laundry must be done by others ............... 0

Travels independently on public transport/own car ............... 1

Travels on public transport when accompanied by others ............... 1

Travel limited to car with assistance from another person ............... 0

Does not travel at all ............... 0

Is capable of taking medicines in correct dosage at correct time .......... 1

Takes medicine if given in separate dosage .................. 0

Is not capable of dispensing own medicines ..................... 0

Manages financial matters independently (budget, cheques, bills) ......... 1

Manages day to day purchases, but need help with banking, etc ............. 1

Incapable of handling money ............ 0

Score from 0-8 was available in the data.

**Self-Rated Health (SRH)**

1. Rate general health condition

The response were:

Excellent........................................................ 1

Very good...................................................... 2

Good .............................................................. 3

Fair................................................................. 4

Poor ............................................................... 5

Good includes (1/3) and poor includes (4/5).

**Types of General Health Questions**

Some questions about your daily life.

1. Recently able to concentrate on whatever doing

2. Recently lost much sleep due to some worry

3. Recently felt constantly under strain

4. Recently felt like couldn’t overcome difficulties

5. Recently been feeling unhappy and depressed?

6. Recently been losing self confidence

7. Recently been thinking self as a worthless person

8. Recently felt like playing a useful role in life

9. Recently felt capable of making decisions about things

10. Recently been able to enjoy normal day-to-day activities

11. Recently been able to face up problems?

12. Recently been feeling reasonably happy, all things considered

The Response were:

Better than usual……….………….…1

Same as usual……………………..…1

Less than usual………………………0

Much less than usual………………...0

Not at all…………………………….1

No more than usual………………….1

Rather more than usual….…………..0

Much more than usual……………….0

Not at all……………………………..1

No more than usual…………………..1

Rather more than usual………………0

Much more than usual……………….0

Not at all…………………………….1

No more than usual………………….1

Rather more than usual………………0

Much more than usual……………….0

Not at all…………………………….1

No more than usual………………….1

Rather more than usual………………0

Much more than usual……………….0

Not at all…………………………….1

No more than usual………………….1

Rather more than usual………………0

Much more than usual……………….0

Not at all…………………………….1

No more than usual………………….1

Rather more than usual………………0

Much more than usual……………….0

More so than usual…………………..1

Same as usual………………………..1

Less useful than usual……………….0

Much less useful…………………….0

More so than usual…………………..1

Same as usual………………………..1

Less capable than usual………………0

Much less capable……………………0

More so than usual…………………..1

Same as usual………………………..1

Less so than usual……………………0

Much less than usual…………………0

More so than usual…………………..1

Same as usual………………………..1

Less able than usual………………….0

Much less useful…………………….0

More so than usual…………………..1

Same as usual………………………..1

Less so than usual……………………0

Much less than usual…………………0

Score of 0-12 was generated and less than five was 1 “Low” and more than equals five as 0 “high”

**Types of Subjective Well being**

How health has been in general over the past few weeks.

1.Feel like life is interesting.

1. Compared with the past, it feels like present life is.

3.On the whole, how happy with the kind of things doing in recent years

4. Achieved the standard of living and the social status in life as expected

5. The extent to which have achieved success and getting ahead

6. Feel like normally accomplished whatever wanted to accomplish

7. Feel like able to manage situations even when they do not turn out to be as expected

8. Feel like confident that in case of crisis (anything that substantially upsets the situation in life), will be able to handle it or face it boldly.

9. With the things going on now, feel confident in coping with future.

The Responses were:

Very much…………………………....1

To some extent………………………..1

Not so much…………………………..0

Very happy………………………….1

Quite happy…………………………1

Not so happy………………………..0

Very happy…………………………..1

Quite happy………………………….1

Not so happy…………………………0

Very much……………………………1

To some extent……………………….1

Not so much………………………….0

Very much……………………………1

To some extent……………………….1

Not so much………………………….0

Most of the time………………………1

Sometimes…………………………….1

Hardly ever……………………………0

Most of the time………………………1

Sometimes…………………………….1

Hardly ever……………………………0

Very much…………………………....1

To some extent………………………..1

Not so much…………………………..0

Very much…………………………....1

To some extent………………………..1

Not so much…………………………..0

Score of 0-9 was generated and less than five was 1 “Low” and more than equals five as 0 “high”

**Cognitive ability**

The words used for testing cognitive impairment were Bus, House, Chair, Banana, Sun, Bird, Cat, Saree, Rice, and Monkey.

Five or more words were recoded as 0 “low” representing lower cognitive impairment and a score of four or less was recoded as 1 “high” representing higher cognitive impairment
